# Salmagundi

**Published:** 1888-05

**Authors:** 


### SALMAGUNDI.

EDITED BY E. G. BETTY, D.D.S.

Antiseptic Mouth Wash.—Such a wash is a most desirable adjunct to the toilet—to say nothing of its value in other respects. The writer for several years past has been prescribing as follows: ℞ Listerine (Lambert's) Tinct. Catechu aa ℥ii Mft. Sig. use thirty to forty drops on a toothbrush (badger is best for the purpose) several times daily and always before retiring for the night. Dr. Miller, of Berlin, has recently indorsed listerine in complimentary terms. In cases of pyorrhoea alveolaris, inflammation of the buccal mucus membrane, apthous stomatitis, cancrum oris, hypertrophy of the gingival margins, etc., the above acts very kindly. The addition of tinct. catechu gives a desirable astringency and furthermore has the quality of being missible with aqueous fluids—in consequence leaving no gummy deposit in pyorrhoeal pockets as does myrrhae and tinct. kino.

The coming meeting of the American Medical Association in Cincinnati, May 8 to 11, bids fair to be a great success as a whole. Modesty forbids our praising before hand the transactions of the Dental Section which we have every assurance will eclipse any previous meeting. The experience at Washington will greatly assist the conduct of this meeting and every one coming may feel that all will be done that can in any way add to the scientific value of the discussions and general conduct of business, and furthermore, all being Americans no distinctions will be made to which any one can take exception. With this thought in mind, we wish it all the success possible and will add our mite to the general fund, in more ways than one.

---

Salol Tooth Powder.—We have recently been making some experiments with salol as the antiseptic element in tooth-powder with some degree of satisfaction. At present the formula is thus:

℞. Salol.................................℈ii gr. v.

Pulv. os lepiac.......................ʒiss

Cretae preparata.....................ʒvi

Magnesia Carbonate..................ʒiv

Pulv. sacch alba.......................ʒiss

Mft. pulvis.

The objection to too much chalk is that it is liable to get wet and cake; too much magnesia makes the powder "fluffy" and in consequence it scatters about freely when charging the brush. The salol has a faint taste of wintergreen.

---

Dr. George S. Meigs, a dentist living in New York, killed himself Jan. 23, 1888, in his operating-room. He had become subject to extreme nervousness, which interfered with his work and kept patients from him. When utterly prostrated, he consented to try the so-called "Christian Science," in the hope that it might restore him to health. It failed, and in despair the wretched man put an end to his life. He made death sure by drinking enough prussic acid to kill twelve men. He stated, in a letter to his wife, that he wished to be cremated.

In our paragraph last month on the "Old Stagers" reproducing themselves in their sons, by some hook or crook, *lapsus pennae* probably, mention should have been made of Dr. W. H. Sedgwick and his son W. H. jr.—both of whom were in attendance at the M. V. D. A.; the two boys, elder and younger, hold up their end of the profession at Granville, O., and never miss an opportunity to attend a good meeting. Come again.

